# Neurodegenerative disease: models, mechanisms, and a new hope

**DOI:** 10.1242/dmm.030205

**Published:** 2017-05-01

**Authors:** Aaron D. Gitler, Paraminder Dhillon, James Shorter

**Affiliations:** 1 Department of Genetics, Stanford University School of Medicine, Stanford, CA 94404, USA; 2 Reviews Editor, Disease Models & Mechanisms, The Company of Biologists, Cambridge CB24 9LF, UK; 3 Department of Biochemistry & Biophysics, Perelman School of Medicine at the University of Pennsylvania, Philadelphia, PA 19104, USA

## Abstract

Neurodegeneration is a feature of many debilitating, incurable diseases that are rapidly rising in prevalence, such as Parkinson's disease. There is an urgent need to develop new and more effective therapeutic strategies to combat these devastating diseases. Models – from cell-based systems, to unicellular organisms, to complex animals – have proven to be a useful tool to help the research community shed light on the mechanisms underlying neurodegenerative diseases, and these advances have now begun to provide promising therapeutic avenues. In this themed issue of Disease Models & Mechanisms, a special collection of articles focused on neurodegenerative diseases is introduced. The collection includes original research articles that provide new insights into the complex pathophysiology of such diseases, revealing candidate biomarkers or therapeutic targets. Some of the articles describe a new disease model that enables deeper exploration of key mechanisms. We also present a series of reviews that highlight some of the recent translational advances made in studies of neurodegenerative diseases. In this Editorial, we summarize the articles featured in this collection, emphasizing the impact that model-based studies have made in this exciting area of research.

## Abstract (precis)

**Summary:** This Editorial introduces a new Special Collection, ‘Neurodegeneration: from models to mechanisms to therapies’, providing a summary of the research and review articles published in this launch issue, as well as highlighting some of DMM's most-read neurodegeneration-related articles from recent issues.

## Introduction

Neurodegenerative diseases represent a major threat to human health. These age-dependent disorders are becoming increasingly prevalent, in part because the elderly population has increased in recent years (Heemels, 2016). Examples of neurodegenerative diseases are Alzheimer's disease, Parkinson's disease, Huntington's disease, amyotrophic lateral sclerosis, frontotemporal dementia and the spinocerebellar ataxias. These diseases are diverse in their pathophysiology – with some causing memory and cognitive impairments and others affecting a person's ability to move, speak and breathe (Abeliovich and Gitler, 2016; Canter et al., 2016; Taylor et al., 2016; Wyss-Coray, 2016). Effective treatments are desperately needed but will only come with a deep understanding of the causes and mechanisms of each disease.

One way to learn about how a disease works is to develop a model system that recapitulates the hallmark characteristics of the disease. Powerful experimental model organisms such as the mouse, fruit fly, nematode worm, and even baker's yeast have been used for many years to study neurodegenerative diseases and have provided key insights into disease mechanisms (Link, 1995; Krobitsch and Lindquist, 2000; Boillee et al., 2006; Bruijn et al., 1998; Yamamoto et al., 2000; Auluck et al., 2002; Outeiro and Lindquist, 2003; Cooper et al., 2006; Gitler et al., 2008, 2009; Elden et al., 2010; Couthouis et al., 2011; Armakola et al., 2012; Jovičić et al., 2015; Becker et al., 2017).

The more recently acquired ability to generate induced-pluripotent stem cells (iPSCs) from differentiated human cells has made it possible to generate patient-specific cell lines in a tissue culture dish – generating human models of human disease (Han et al., 2011). Recently, there have been technological innovations that allow for these cells to be cultured in three dimensions, to produce organoids that represent various human tissues, even the brain (Marton and Paşca, 2016; Paşca et al., 2015). These three-dimensional brain organoid systems permit cell-cell interactions and complex cyto-architecture to be modeled and studied in greater detail and in more physiological contexts than traditional tissue culture models with isolated cells. Furthermore, accruing evidence suggests that many neurodegenerative diseases are not merely diseases of dying neurons. Non-neuronal cells in the brain, such as glial cells, which are even more abundant in the brain and the central nervous system than neurons, play major roles in disease progression (Ilieva et al., 2009). Incorporating these neuron-glial interactions into such 3D brain organoid models will empower the elucidation of cell non-autonomous disease mechanisms. We anticipate that these 3D brain organoid systems will be a powerful addition to the disease modeler's experimental arsenal. Remarkable advances in genome sequencing technology have made it possible to read genomes of individual patients to discover causes of both rare and common genetic diseases. But which sequence variants are damaging to gene function and which are benign? Model organisms will also be powerful tools to test the effects of candidate gene variants discovered by human genome sequencing.

One highly inspirational success story is the development of a therapy for spinal muscular atrophy (SMA). SMA – a neuromuscular disease caused by loss-of-function mutations in the *SMN1* gene – is the most common genetic killer of babies. Pioneering studies of the molecular mechanisms of the disease and the development of animal models (Hua et al., 2010, 2011) laid the foundation for the recent clinical trials testing antisense oligonucleotides (ASOs) as a therapeutic strategy to correct a splicing defect and restore functional SMN protein. Studies in animal model systems revealed that this therapeutic strategy could work (Hua et al., 2010, 2011) and two recent clinical trials in children with SMA demonstrated that the strategy does work. In a spectacular advance, infants that received the ASO drug showed substantial improvement in motor function compared with children who did not receive the drug (Finkel et al., 2016). At the end of 2016, the United States Food and Drug Administration approved this therapy, making it the first disease-modifying treatment for SMA. A remarkable and game-changing win for model systems and especially for patients and their families. These stunning results augur well for ASO drugs that are being developed for testing in several other neurodegenerative diseases, including Huntington's disease and amyotrophic lateral sclerosis (ALS). We now have a new hope and a clear path forward for effective therapies for neurodegenerative diseases. It truly is an inspiring and hopeful time to be a researcher in this field.

Our goal in compiling this special Disease Models & Mechanisms (DMM) collection, ‘Neurodegeneration: From Models to Mechanisms to Therapies’, is to convey the sense of excitement and hope in the neurodegenerative disease research field as well as to acknowledge the challenges ahead. To launch the collection, we present, in this issue, a number of new reviews and research articles that demonstrate the translational impact of studies involving neurodegenerative disease model systems. The collection also includes some of DMM's most-read neurodegeneration-focused research and review content from recent years. Below, we summarize the collection so far and provide a preview of what's to come in upcoming issues of the journal.

## Reviews: trends in neurodegeneration research

We begin this neurodegeneration-focused issue with an exclusive interview with Huda Zoghbi, a professor in the Departments of Pediatrics, Molecular and Human Genetics, Neurology and Neuroscience at Baylor College of Medicine. Huda is renowned for her many groundbreaking discoveries in neurological disease research, not least her contributions to elucidating the genetic causes and molecular mechanisms underlying spinocerebellar ataxia and Rett syndrome. These insights have also shed light on the complex mechanisms at play in common neurodegenerative diseases. In this interview, Huda describes her journey from the clinic to the bench, highlighting the experiences, mentors and collaborations that helped to shape her research interests (Zoghbi, 2017). One of the founding editors of DMM, Huda has always been an advocate of the use of model systems in human disease research, and her interview particularly emphasizes the power of animal models for exploring brain disorders. Previous DMM ‘A Model for Life’ interviewees whose research is also focused on neurodegenerative diseases include David Rubinsztein and Rick Morimoto (all interviews can be found at: http://dmm.biologists.org/content/by/section/A MODEL FOR LIFE).

Next, in the review section of this issue, Edward Lee and colleagues present an ‘At a Glance’ overview of RNA metabolism in neurodegenerative diseases (Liu et al., 2017). In recent years, altered RNA processing has emerged – largely through studies involving model systems – as a key contributing factor in several neurodegenerative diseases. This article and its accompanying poster illustrate how impaired RNA metabolism can underlie the pathophysiology of neurodegeneration, as well as discussing potential therapeutic interventions to target these processes. An earlier DMM ‘At a Glance’ poster, by Julie Valastyan and Susan Lindquist, provides a snapshot of protein-level mechanisms that have long been implicated in neurodegenerative and related disorders (Valastyan and Lindquist, 2014).

Another of the new reviews published in this issue focuses in on the role of calcium signaling in Parkinson's disease. As a major global health problem, Parkinson's disease has been the focus of a high proportion of model-based research in recent years, and many such studies have indicated that disruption of calcium homeostasis is a key pathological feature. This review, written by Gabriela Caraveo and colleagues, discusses the current evidence for dysregulation of calcium-dependent pathways in this disease and potential for these findings to guide drug development (Zaichick et al., 2017).

Rounding up the review section, Wim Robberecht and colleagues provide a comprehensive discussion of model systems used to study ALS, from cell-based systems to fruit flies to rodents (Van Damme et al., 2017). Thanks to the diverse range of models available to study this devastating disease – emphasized by the many ALS-related research papers also published in this issue (see below) – many of the genes and pathways involved have been unveiled. The ALS research community is now poised to translate these findings to the clinic. A related DMM review published in 2014 highlighted the impact that zebrafish models in particular have made in improving our understanding of the pathogenic mechanisms underpinning ALS and related motor neuron disorders (Patten et al., 2014).

## New research: models, mechanisms and more

In addition to the review content, this Special Collection showcases original research articles that impact our understanding of neurodegenerative disease mechanisms. These papers span different diseases, from common ones such as Parkinson's disease and Alzheimer's disease to rare neurological disorders. Some focus on modeling human gene defects in animals, whereas others develop biomarkers to track disease progression. In each case, they show how diverse model systems can be powerfully used to explore the mechanisms of neurodegeneration. Below, we highlight the key findings and translational implications of a subset of the new research papers published in this issue.

In the first of ten new research papers, Javier Fernández-Ruiz and colleagues extend previously published observations from a mouse model of familial ALS to a canine model of the disease (Fernández-Trapero et al., 2017). Dogs can develop a type of neurodegenerative disorder called degenerative myelopathy, which can be caused by mutations in the same gene that is frequently mutated in human ALS: superoxide dismutase 1 (*SOD1*). The observation that cannabinoid receptor type-2 (CB_2_) expression is upregulated in human ALS and in a mouse model of ALS caused by *SOD1* mutations led the authors to test for similar effects in the dog model. Consistent with previous results, they found that CB_2_ receptor expression is upregulated in the spinal cord of dogs affected by degenerative myelopathy (Fernández-Trapero et al., 2017). These results lend weight to the putative neuroprotective effect of CB_2_ receptor modulation in ALS, and further support the use of canine degenerative myelopathy as a relevant model to study the pathophysiology of this disease. The dog model represents the first spontaneous animal model of ALS, offering several advantages over models that rely on transgenic overexpression.

Also in this issue, Paulo Ferreira and colleagues contribute to the exciting new concept that impairments in nucleocytoplasmic transport can underlie neurodegenerative diseases (Cho et al., 2017). Using a conditional knockout approach in mice, the authors inactivated RANBP2, a regulator of the Ran GTPase cycle, which is known to power nuclear import. Selective loss of RANBP2 function from motor neurons led to ALS-like symptoms, including progressive neuron loss, weakness, and fatal paralysis. At the cellular level, nucleocytoplasmic transport and proteostasis of substrates involved in motor neuron homeostasis were disrupted, supporting the involvement of the Ran GTPase cycle in ALS pathophysiology. The new conditional mouse model generated here can be used for further mechanistic exploration (Cho et al., 2017).

Loss of appetite and weight loss are common features of neurodegenerative diseases such as ALS. In another new paper, Sabine Cordes and colleagues use mouse genetics to explore the molecular basis for regulating appetite and focus their studies on a role of the tyrosine receptor kinase 3 (*Tyro3*) gene in regulating the neural circuitry that governs appetite (Kim et al., 2017). They provide evidence that *Tyro3* can act as a modifier for the *anorexia* mutation in mouse. In addition to shedding light on the neuropathological basis of anorexia, their findings could help suggest strategies to prevent or slow weight loss that accompanies neurodegenerative disorders.

The neuromuscular junction is a remarkable structure that is particularly vulnerable to neurodegenerative disease. However, it possesses powerful ways to resist injury and to regenerate. In their new DMM paper, Michela Rigoni and colleagues use an *in vitro* cellular model of the degenerative disease Miller Fisher syndrome to define the Schwann cell and neuron signaling events, components and cross-talk required to promote nerve regeneration in the face of peripheral neuropathies (Rodella et al., 2017).

The peripheral nervous system (PNS) is also a target of neurodegenerative disease. A group of disorders called hereditary sensory and autonomic neuropathies (HSANs) are caused by PNS dysfunction. One such disorder, familial dysautonomia, is caused by mutation of the *IKBKAP* gene. Reported in this issue, Frances Lefcort and colleagues generate the first mouse model to study the consequence of loss of *IKBKAP* specifically in neurons (Chaverra et al., 2017). The mutant mice develop both autonomic and non-autonomic neuronal deficits, suggesting a role for *IKBKAP* beyond the PNS, to CNS development and function.

Parkinson's disease (PD) is one of the most common age-related neurodegenerative disorders worldwide. The ability to predict and detect the disease presymptomatically on the basis of biomarkers is likely to give therapies the best chance of working. Non-invasive biomarkers, such as blood or imaging-based markers are ideal, because they can be analyzed longitudinally and could even be used to track the efficacy of a proposed treatment. In one of two new PD-focused studies in this issue, Georg Auburger and colleagues analyzed the blood samples of a large Turkish family affected by a PD-causing duplication of the α-synuclein gene (*SNCA*) (Lahut et al., 2017). They detected significant downregulation of complexin-1 (*CPLX1*) mRNA in the blood of presymptomatic heterozygotes at risk of PD based on prodromal features, and performed functional studies to provide insight into the role of CPLX1 in PD. Their study provides a new blood biomarker with the potential to be used in early screens for PD risk.

Defining intrinsic cellular defects that characterize a particular disease state can provide mechanistic insight but could also serve as a biomarker to predict disease based on analysis of patient cells. Miguel Weil and colleagues discovered that human mesenchymal stem cells (hMSCs) isolated from the bone marrow of sporadic ALS patients have an altered response to DNA-damaging agents compared with hMSCs from healthy subjects (Wald-Altman et al., 2017). The authors connect this defect to autophagy and show that autophagy regulation is perturbed in ALS-patient cells. These findings reinforce a role for autophagy dysregulation in ALS and also provide a potential cellular biomarker of the disease.

Last but not least, Laura Calzà and colleagues examined the vulnerability of neurons from a transgenic mouse model of Alzheimer's disease (AD) to oxygen-glucose deprivation (an *in vitro* model of hypoxia) (Baldassarro et al., 2017). This is an important question in AD research as vascular dysfunction is a feature of the disease, but the link with amyloid pathology remains unclear. Calzà and co-authors demonstrate that intraneuronal amyloid-β (Aβ) accumulation in neurons increases vulnerability to cell death upon oxygen-glucose deprivation, and interestingly, exposure of neurons to astrocyte-conditioned culture media confers protection. These results highlight important cell non-autonomous interactions between neurons and glia in Alzheimer's disease.

## Greatest hits

Beyond the papers featured in this thematic issue, there have been several exciting papers focused on neurodegeneration published in *DMM* in the past year. We cannot highlight all of them because of space limitations, but we would like to mention a couple of standout papers from 2016. First, in a study examining the convoluted issue of mitochondrial dysfynction in PD, Paul Fisher and colleagues reported that patient-derived immortalized lymphoblasts show enhanced respiratory activity compared with control cells (Annesley et al., 2016). The authors propose that this trait could provide a reliable biomarker of the disease.

The second study of note is by Ellen Sidransky and colleagues, and focuses on Gaucher disease. Mutation of *GBA1*, which encodes the lysosomal enzyme glucocerebrosidase, causes Gaucher disease. In one of the most exciting recent developments in the neurodegenerative disease field in the last several years, it has been appreciated that mutations in *GBA1* are also a risk factor for Parkinson's disease. How Gaucher disease connects to Parkinson disease at the molecular and cellular level is an area of intense interest (Abeliovich and Gitler, 2016). Sidransky and her colleagues have generated a mouse neuronal cell model with nearly complete loss of glucocerebrosidase activity, mimicking the situation in Gaucher disease (Westbroek et al., 2016). This model provides the field a powerful tool to study not only Gaucher disease but also to explore potential connections with PD pathophysiology. Keep an eye out for a review by Sidransky and co-authors on iPSC models of lysosomal storage disorders in an upcoming issue of the journal.

## Concluding remarks

We hope you enjoy this issue and the overall collection, which will be added to over the coming months. All articles can be freely accessed via this dedicated page: http://dmm.biologists.org/collection/neurodegenerative-disorders. We dedicate this collection to Susan Lindquist, our beloved mentor, friend, and founding editor of DMM who sadly passed away in October, 2016. Among several moving tributes (Chernoff, 2017; Serio, 2017; Bevis, 2017; Morimoto, 2016; Hartl, 2016; Shorter and Gitler, 2016; Whitesell and Santagata, 2016; Fuchs, 2016; Khurana et al., 2017), a particularly touching obituary written by Vivian Siegel was published in DMM (Siegel, 2017). Sue was a visionary pioneer who was unsurpassed in her ability to harness model systems to explore fundamental biology and elucidate mechanisms of human disease. Her fearlessness led her to champion the humble budding yeast as a simple, yet powerful new model to study the cell biology underpinning neurodegeneration. This pioneering strategy allowed Sue to quickly arrive at mechanism, which could then be rapidly translated to more complex model systems. We suspect that these insights will soon reach the clinic.

## References

[DMM030205C1] Abeliovich A. and Gitler A. D. (2016). Defects in trafficking bridge Parkinson's disease pathology and genetics. *Nature* 539, 207-216. 10.1038/nature20414 27830778

[DMM030205C2] Annesley S. J., Lay S. T., De Piazza S. W., Sanislav O., Hammersley E., Allan C. Y., Francione L. M., Bui M. Q., Chen Z.-P., Ngoei K. R. W. et al. (2016). Immortalized Parkinson's disease lymphocytes have enhanced mitochondrial respiratory activity. *Dis. Model. Mech.* 9, 1295-1305. 10.1242/dmm.025684 27638668 PMC5117226

[DMM030205C3] Armakola M., Higgins M. J., Figley M. D., Barmada S. J., Scarborough E. A., Diaz Z., Fang X., Shorter J., Krogan N. J., Finkbeiner S. et al. (2012). Inhibition of RNA lariat debranching enzyme suppresses TDP-43 toxicity in ALS disease models. *Nat. Genet.* 44, 1302-1309. 10.1038/ng.2434 23104007 PMC3510335

[DMM030205C4] Auluck P. K., Chan H. Y., Trojanowski J. Q., Lee V. M. and Bonini N. M. (2002). Chaperone suppression of alpha-synuclein toxicity in a Drosophila model for Parkinson's disease. *Science* 295, 865-868. 10.1126/science.1067389 11823645

[DMM030205C5] Baldassarro V. A., Marchesini A., Giardino L. and Calzà L. (2017). Vulnerability of primary neurons derived from Tg2576 Alzheimer mice to oxygen and glucose deprivation: role of intraneuronal amyloid-β accumulation and astrocytes. *Dis. Model. Mech.* 10, 671-678. 10.1242/dmm.028001 28237964 PMC5451168

[DMM030205C6] Becker L. A., Huang B., Bieri G., Ma R., Knowles D. A., Jafar-Nejad J., Messing J., Kim H. J., Soriano A., Auburger G. et al. (2017). Therapeutic reduction of ataxin 2 extends lifespan and reduces pathology in TDP-43 mice. *Nature* 544, 367-371. 10.1038/nature22038 28405022 PMC5642042

[DMM030205C7] Bevis B. J. (2017). Susan Lindquist: visionary scientist and peerless mentor. *J. Cell Biol.* 216, 5-8. 10.1083/jcb.201612112 28028126 PMC5223621

[DMM030205C8] Boillee S., Yamanaka K., Lobsiger C. S., Copeland N. G., Jenkins N. A., Kassiotis G., Kollias G. and Cleveland D. W. (2006). Onset and progression in inherited ALS determined by motor neurons and microglia. *Science* 312, 1389-1392. 10.1126/science.1123511 16741123

[DMM030205C9] Bruijn L. I., Houseweart M. K., Kato S., Anderson K. L., Anderson S. D., Ohama E., Reaume A. G., Scott R. W. and Cleveland D. W. (1998). Aggregation and motor neuron toxicity of an ALS-linked SOD1 mutant independent from wild-type SOD1. *Science* 281, 1851-1854. 10.1126/science.281.5384.1851 9743498

[DMM030205C10] Canter R. G., Penney J. and Tsai L.-H. (2016). The road to restoring neural circuits for the treatment of Alzheimer's disease. *Nature* 539, 187-196. 10.1038/nature20412 27830780

[DMM030205C11] Chaverra M., George L., Mergy M., Waller H., Kujawa K., Murnion C., Sharples E., Thorne J., Podgajny N., Grindeland A. et al. (2017). The familial dysautonomia disease gene *IKBKAP* is required in the developing and adult mouse central nervous system. *Dis. Model. Mech*. 10, 605-618. 10.1242/dmm.028258 28167615 PMC5451171

[DMM030205C12] Chernoff Y. O. (2017). In memory of Susan Lindquist (1949-2016). *Prion* 11, 1-3. 10.1080/19336896.2017.1285618 28281923 PMC5360134

[DMM030205C13] Cho K.-i., Yoon D., Qiu S., Danziger Z., Grill W. M., Wetsel W. C. and Ferreira P. A. (2017). Loss of Ranbp2 in motoneurons causes disruption of nucleocytoplasmic and chemokine signaling, proteostasis of hnRNPH3 and Mmp28, and development of amyotrophic lateral sclerosis-like syndromes. *Dis. Model. Mech*. 10, 559-579. 10.1242/dmm.027730 28100513 PMC5451164

[DMM030205C14] Cooper A. A., Gitler A. D., Cashikar A., Haynes C. M., Hill K. J., Bhullar B., Liu K., Xu K., Strathearn K. E., Liu F. et al. (2006). Alpha-synuclein blocks ER-Golgi traffic and Rab1 rescues neuron loss in Parkinson's models. *Science* 313, 324-328. 10.1126/science.1129462 16794039 PMC1983366

[DMM030205C15] Couthouis J., Hart M. P., Shorter J., Dejesus-Hernandez M., Erion R., Oristano R., Liu A. X., Ramos D., Jethava N., Hosangadi D. et al. (2011). A yeast functional screen predicts new candidate ALS disease genes. *Proc. Natl. Acad. Sci. USA* 108, 20881-20890. 10.1073/pnas.1109434108 22065782 PMC3248518

[DMM030205C16] Elden A. C., Kim H.-J., Hart M. P., Chen-Plotkin A. S., Johnson B. S., Fang X., Armakola M., Geser F., Greene R., Lu M. M. et al. (2010). Ataxin-2 intermediate-length polyglutamine expansions are associated with increased risk for ALS. *Nature* 466, 1069-1075. 10.1038/nature09320 20740007 PMC2965417

[DMM030205C17] Fernández-Trapero M., Espejo-Porras F., Rodríguez-Cueto C., Coates J. R., Pérez-Díaz C., De Lago E. and Fernández-Ruiz J. (2017). Upregulation of CB_2_ receptors in reactive astrocytes in canine degenerative myelopathy, a disease model of amyotrophic lateral sclerosis. *Dis. Model. Mech*. 10, 551-558. 10.1242/dmm.028373 28069688 PMC5451172

[DMM030205C18] Finkel R. S., Chiriboga C. A., Vajsar J., Day J. W., Montes J., De Vivo D. C., Yamashita M., Rigo F., Hung G., Schneider E. et al. (2016). Treatment of infantile-onset spinal muscular atrophy with nusinersen: a phase 2, open-label, dose-escalation study. *Lancet* 388, 3017-3026. 10.1016/S0140-6736(16)31408-8 27939059

[DMM030205C19] Fuchs E. (2016). Susan Lee Lindquist (1949–2016). *Cell* 167, 1440-1442. 10.1016/j.cell.2016.11.030 29413691

[DMM030205C20] Gitler A. D., Bevis B. J., Shorter J., Strathearn K. E., Hamamichi S., Su L. J., Caldwell K. A., Caldwell G. A., Rochet J.-C., Mccaffery J. M. et al. (2008). The Parkinson's disease protein alpha-synuclein disrupts cellular Rab homeostasis. *Proc. Natl. Acad. Sci. USA* 105, 145-150. 10.1073/pnas.0710685105 18162536 PMC2224176

[DMM030205C21] Gitler A. D., Chesi A., Geddie M. L., Strathearn K. E., Hamamichi S., Hill K. J., Caldwell K. A., Caldwell G. A., Cooper A. A., Rochet J. C. et al. (2009). Alpha-synuclein is part of a diverse and highly conserved interaction network that includes PARK9 and manganese toxicity. *Nat. Genet.* 41, 308-315. 10.1038/ng.300 19182805 PMC2683786

[DMM030205C22] Han S. S. W., Williams L. A. and Eggan K. C. (2011). Constructing and deconstructing stem cell models of neurological disease. *Neuron* 70, 626-644. 10.1016/j.neuron.2011.05.003 21609821

[DMM030205C23] Hartl F. U. (2016). Susan Lee Lindquist (1949-2016)-pioneer in the study of cellular protein folding and disease. *EMBO J.* 35, 2626-2627. 10.15252/embj.201670040 27920028 PMC5167337

[DMM030205C24] Heemels M.-T. (2016). Neurodegenerative diseases. *Nature* 539, 179 10.1038/539179a 27830810

[DMM030205C25] Hua Y., Sahashi K., Hung G., Rigo F., Passini M. A., Bennett C. F. and Krainer A. R. (2010). Antisense correction of SMN2 splicing in the CNS rescues necrosis in a type III SMA mouse model. *Genes Dev.* 24, 1634-1644. 10.1101/gad.1941310 20624852 PMC2912561

[DMM030205C26] Hua Y., Sahashi K., Rigo F., Hung G., Horev G., Bennett C. F. and Krainer A. R. (2011). Peripheral SMN restoration is essential for long-term rescue of a severe spinal muscular atrophy mouse model. *Nature* 478, 123-126. 10.1038/nature10485 21979052 PMC3191865

[DMM030205C27] Ilieva H., Polymenidou M. and Cleveland D. W. (2009). Non-cell autonomous toxicity in neurodegenerative disorders: ALS and beyond. *J. Cell Biol.* 187, 761-772. 10.1083/jcb.200908164 19951898 PMC2806318

[DMM030205C28] Jovičić A., Mertens J. Boeynaems S., Bogaert E., Chai N., Yamada S. B., Paul J. W., 3rd, Sun S., Herdy J. R., Bieri G., Kramer N. J., Gage F. H., Van Den Bosch L., Robberecht W. and Gitler A. D. (2015). Modifiers of *C9orf72* dipeptide repeat toxicity connect nucleocytoplasmic transport defects to FTD/ALS. *Nat. Neurosci.* 18, 1226-1229. 10.1038/nn.4085 26308983 PMC4552077

[DMM030205C29] Khurana V., Chung C. Y. and Tardiff D. F. (2017). From yeast to patients: the audacity and vision of Susan Lindquist. *Cell Syst.* 4, 147-148. 10.1016/j.cels.2017.02.007 28231447

[DMM030205C30] Kim D. Y., Yu J., Mui R. K., Niibori R., Taufique H. B., Aslam R., Semple J. W. and Cordes S. P. (2017). The tyrosine kinase receptor Tyro3 enhances lifespan and neuropeptide Y (Npy) neuron survival in the mouse anorexia (*anx*) mutation. *Dis. Model. Mech*. 10, 581-595. 10.1242/dmm.027433 28093506 PMC5451163

[DMM030205C31] Krobitsch S. and Lindquist S. (2000). Aggregation of huntingtin in yeast varies with the length of the polyglutamine expansion and the expression of chaperone proteins. *Proc. Natl. Acad. Sci. USA* 97, 1589-1594. 10.1073/pnas.97.4.1589 10677504 PMC26479

[DMM030205C32] Lahut S., Gispert S., Ömür Ö., Depboylu C., Seidel K., Dominguez-Bautista J. A., Brehm N., Tireli H., Hackmann K., Pirkevi C. et al. (2017). Blood RNA biomarkers in prodromal PARK4 and REM sleep behavior disorder show role of complexin-1 loss for risk of Parkinson's disease. *Dis. Model. Mech*. 10, 619-631. 10.1242/dmm.028035 28108469 PMC5451169

[DMM030205C33] Link C. D. (1995). Expression of human beta-amyloid peptide in transgenic *Caenorhabditis elegans*. *Proc. Natl. Acad. Sci. USA* 92, 9368-9372. 10.1073/pnas.92.20.9368 7568134 PMC40986

[DMM030205C34] Liu E. Y., Cali C. P. and Lee E. B. (2017). RNA metabolism in neurodegenerative disease. *Dis. Model. Mech*. 10, 509-518. 10.1242/dmm.028613 28468937 PMC5451173

[DMM030205C35] Marton R. M. and Paşca S. P. (2016). Neural differentiation in the third dimension: generating a human midbrain. *Cell Stem Cell* 19, 145-146. 10.1016/j.stem.2016.07.017 27494668

[DMM030205C36] Morimoto R. I. (2016). Susan Lindquist 1949-2016. *Nat. Struct. Mol. Biol.* 23, 1072-1073. 10.1038/nsmb.3339 27922616

[DMM030205C37] Outeiro T. F. and Lindquist S. (2003). Yeast cells provide insight into alpha-synuclein biology and pathobiology. *Science* 302, 1772-1775. 10.1126/science.1090439 14657500 PMC1780172

[DMM030205C38] Paşca A. M., Sloan S. A., Clarke L. E., Tian Y., Makinson C. D., Huber N., Kim C. H., Park J.-Y., O'rourke N. A., Nguyen K. D. et al. (2015). Functional cortical neurons and astrocytes from human pluripotent stem cells in 3D culture. *Nat. Methods* 12, 671-678. 10.1038/nmeth.3415 26005811 PMC4489980

[DMM030205C39] Patten S. A., Armstrong G. A. B., Lissouba A., Kabashi E., Parker J. A. and Drapeau P. (2014). Fishing for causes and cures of motor neuron disorders. *Dis. Mod. Mech.* 7, 799-809. 10.1242/dmm.015719 PMC4073270 24973750

[DMM030205C40] Rodella U., Negro S., Scorzeto M., Bergamin E., Jalink K., Montecucco C., Yuki N. and Rigoni M. (2017). Schwann cells are activated by ATP released from neurons in an in vitro cellular model of Miller Fisher syndrome. *Dis. Model. Mech*. 10, 597-603. 10.1242/dmm.027870 28067631 PMC5451166

[DMM030205C41] Serio T. R. (2017). Susan Lindquist (1949-2016). *Nat. Chem. Biol.* 13, 127 10.1038/nchembio.2305 28103217

[DMM030205C42] Shorter J. and Gitler A. D. (2016). Susan Lee Lindquist (1949-2016). *Nature* 540, 40 10.1038/540040a 27905439

[DMM030205C43] Siegel V. (2017). Susan Lindquist: a tribute. *Dis. Model. Mech.* 10, 1-2. 10.1242/dmm.028696 28067627 PMC5278528

[DMM030205C44] Taylor J. P., Brown R. H. Jr and Cleveland D. W. (2016). Decoding ALS: from genes to mechanism. *Nature* 539, 197-206. 10.1038/nature20413 27830784 PMC5585017

[DMM030205C45] Valastyan J. S. and Lindquist S. (2014). Susan Lindquist Mechanisms of protein-folding diseases at a glance. *Dis. Model. Mech.* 7, 9-14. 10.1242/dmm.013474 24396149 PMC3882043

[DMM030205C46] Van Damme P., Robberecht W. and Van Den Bosch L. (2017). Modelling amyotrophic lateral sclerosis: progress and possibilities. *Dis. Model. Mech*. 10, 537-549. 10.1242/dmm.029058 28468939 PMC5451175

[DMM030205C47] Wald-Altman S., Pichinuk E., Kakhlon O. and Weil M. (2017). A differential autophagy-dependent response to DNA double-strand breaks in bone marrow mesenchymal stem cells from sporadic ALS patients. *Dis. Model. Mech*. 10, 645-654. 10.1242/dmm.027938 28213588 PMC5451167

[DMM030205C48] Westbroek W., Nguyen M., Siebert M., Lindstrom T., Burnett R. A., Aflaki E., Jung O., Tamargo R., Rodriguez-Gil J. L., Acosta W. et al. (2016). A new glucocerebrosidase-deficient neuronal cell model provides a tool to probe pathophysiology and therapeutics for Gaucher disease. *Dis. Model. Mech* 9, 769-778. 10.1242/dmm.024588 27482815 PMC4958308

[DMM030205C49] Whitesell L. and Santagata S. (2016). Susan Lindquist (1949-2016). *Science* 354, 974 10.1126/science.aal3609 27884995

[DMM030205C50] Wyss-Coray T. (2016). Ageing, neurodegeneration and brain rejuvenation. *Nature* 539, 180-186. 10.1038/nature20411 27830812 PMC5172605

[DMM030205C51] Yamamoto A., Lucas J. J. and Hen R. (2000). Reversal of neuropathology and motor dysfunction in a conditional model of Huntington's disease. *Cell* 101, 57-66. 10.1016/S0092-8674(00)80623-6 10778856

[DMM030205C52] Zaichick S. V., McGrath K. M. and Caraveo G. (2017). The role of Ca^2+^ signaling in Parkinson's disease. *Dis. Model. Mech*. 10, 519-535. 10.1242/dmm.028738 28468938 PMC5451174

[DMM030205C53] Zoghbi H. Y. (2017). Solving the puzzle of neurological diseases: an interview with Huda Zoghbi. *Dis. Model. Mech*. 10, 503-507. 10.1242/dmm.029751 28468936 PMC5451176

